# Economic evaluation of a web-based menu planning intervention to improve childcare service adherence with dietary guidelines

**DOI:** 10.1186/s13012-020-01068-x

**Published:** 2021-01-07

**Authors:** Penny Reeves, Kim Edmunds, Zoe Szewczyk, Alice Grady, Sze Lin Yoong, Luke Wolfenden, Rebecca Wyse, Meghan Finch, Fiona Stacey, John Wiggers, Andrew Searles

**Affiliations:** 1 Hunter Medical Research Institute (HMRI), New Lambton, New South Wales Australia; 2 School of Medicine and Public health, University of Newcastle, Callaghan, New South Wales 2308 Australia; 3 Hunter New England Population Health, Wallsend, New South Wales 2287 Australia; 4 Priority Research Centre for Health Behaviour, University of Newcastle, Callaghan, NSW Australia

**Keywords:** Cost-consequence, Economic evaluation, Dietary intervention, Childhood obesity prevention

## Abstract

**Background:**

Despite the known benefits of healthy eating in childhood, few Australian childcare services provide food that is consistent with dietary guidelines. The effectiveness of a web-based menu planning intervention to increase childcare service provision of healthy foods and decrease provision of discretionary foods in long day-care services in Australia was assessed in a randomised controlled trial. Here we consider the costs, consequences, cost-effectiveness and budget impact of the intervention using data collected within the trial.

**Methods:**

The prospective trial-based economic evaluation involved 54 childcare services across New South Wales (NSW), Australia. Services were randomised to a 12-month intervention or usual care. The intervention involved access to a web-based menu planning and decision support tool and online resources. Effectiveness measures included mean number of food groups, overall menu and individual food group compliance with dietary guidelines, and mean servings of food groups at 12 months. Costs (reported in $AUD, 2017/18) were evaluated from both health sector and societal perspectives. The direct cost to support uptake of the intervention was calculated, as were costs to each childcare service. The incremental cost of the intervention was calculated as the net difference in the cost to undertake menu planning and review plus the direct cost of the intervention. Incremental cost-effectiveness ratios (ICERs) including uncertainty intervals were estimated for differences in costs and effects between intervention and control groups. A relative value index was calculated to determine overall value for money.

**Results:**

Over the 12 months of the trial, we calculated a difference in cost between usual practice and intervention groups of − $482 (95% UI − $859, − $56). While the measured increase in menu and food group compliance within the trial did not reach statistical significance, there were significant improvements in mean servings of fruit and discretionary food, represented in the cost-consequence analysis. The calculated relative value index of 1.1 suggests that the intervention returns acceptable value for money for the outcomes generated.

**Conclusion:**

Compared to usual practice, web-based programmes may offer an efficient and sustainable alternative for childcare services to improve the provision of healthy foods to children in their care.

**Trial registration:**

Australian New Zealand Clinical Trials Registry ACTRN12616000974404

Contributions to the literature

- Research has shown that there is very limited health economic evaluation applied to public health implementation-interventions, potentially resulting in underinvestment in health promoting programmes.
- We found there is value to both decision makers and researchers in measuring and valuing costs even when trial outcome results are modest.
- These findings contribute to recognised gaps in the literature, including the measurement of the opportunity cost of implementation-interventions and the value of economic evidence to support decision-making in health.

## Background

Obesity in early childhood, defined by the World Health Organisation (WHO) as 0 to 8 years of age [1], is associated with a range of adverse social and health outcomes for children and the community. In 2016, the WHO estimated the number of overweight children under the age of five to be over 41 million [2]. Childhood obesity also represents a significant economic burden. In a recent review, Finkelstein and colleagues estimated the incremental lifetime per capita medical cost of an obese child in the USA relative to a normal weight child to be $12,660 to $19,630 (2012 USD) [3]. An Australian study estimated the direct healthcare costs of children with obesity aged 2–4 years to be 1.62 (95% CI 1.12–2.34, *p* = 0.01) times those of healthy weight children [4, 5]. The annual direct costs to the Australian healthcare system of early childhood obesity was calculated to be around $18 million ($2018 AUD) [4]. Two paediatric simulation studies in Germany [6, 7] considered the economic impact of childhood overweight and obesity, estimating total lifetime costs of €1.8 billion (2010) for the current prevalent population.

There are strong economic arguments for targeting early childhood obesity to reduce the economic and social burden of obesity-related chronic disease across the lifespan. Dietary behaviour is a key driver of unhealthy weight gain in children [8]. Since dietary behaviours from early childhood are known to track into adulthood [9, 10], it is recommended that population health initiatives target improvement in the healthy eating behaviours of young children to prevent the onset of obesity [2].

On average across Organisation for Economic Co-operation and Development (OECD) countries, around 33% of children aged 0–2 are enrolled in early childhood education and care services. For children aged 3–5 years, the enrolment rates are generally higher. In the majority of OECD countries, over 80% of children aged 3–5 are enrolled in early childhood education and care or primary school, with an average enrolment rate of 86.3% [11]. In Australia, approximately 61% of Australian children aged 0–5 years attend a service-based form of early childhood education and care [12]. These data suggest that childcare services are an appropriate setting for interventions designed to influence healthy eating behaviours in childhood, given the access they have to large numbers of children aged under 5 years, coupled with children consuming a substantial proportion of their overall energy intake in these settings [13]. In Australia, the provision of food in early childhood services is regulated and should be provided consistent with national dietary guidelines. However, international and Australian research has found that amongst services that provide food, menu dietary guidelines are poorly implemented [14–16]. Improving the implementation of dietary guidelines in childcare services represents a considerable challenge. Previous trials testing implementation support strategies such as the provision of educational materials, face-to-face meetings and/or audit and feedback for childcare cooks have demonstrated variable improvements in guideline adherence [15, 17–20]. In the context of resource constraints and escalating healthcare costs, interventions need not be merely effective. They also need to be cost-effective and affordable to allow policy makers to select value-based programmes to be disseminated at scale. Previous interventions have been labour intensive involving significant face-to-face support and dietitian-led assessment of menu compliance [18, 21–23]. For this reason, web-based interventions are likely to deliver greater value, if proven effective.

Recent systematic reviews have shown that economic evaluation is rarely applied to public health implementation-interventions, and the generation of economic evidence has been identified as a priority for the field of implementation research [13, 24, 25]. Investment in implementation has an opportunity cost. That is, the resources could be put to another use, the value of which is given up. To ensure the efficient and equitable allocation of resources, as well as affordability, it is important to include measures of cost, alongside analysis of intervention consequences [26]. Cost-consequence analysis (CCA) has been recommended for complex public health interventions, such as implementation interventions, which are difficult to measure in a common outcome unit [26, 27]. CCA is differentiated from cost-benefit analysis or cost-utility analysis in that it does not attempt to summarise outcomes in financial terms or in a single metric such as the quality-adjusted life year (quality-adjusted life year). Rather, outcomes are shown in their natural units, and decision makers can determine for themselves whether the intervention is worth the investment [28]. The disadvantage is that without a clear decision rule, value determination is subjective and may lack transparency. Cost-effectiveness analyses (CEA) compare costs with natural units such as life years, number of foods served or number of cases averted, to provide an estimate of economic efficiency [29]. Providing these data enables comparison between interventions and allows decision makers to select strategies that will have the greatest impact given available resources [29]. To address gaps in the economic evidence, an economic evaluation was conducted alongside a randomised controlled trial of a web-based intervention to improve childcare service implementation of dietary guidelines. The economic evaluation was conducted with the dual aim of providing a valuation of the investment required to execute implementation-interventions in community settings and also to generate economic evidence analysis to permit a determination of value for money and inform the cost to scale-up the intervention. Both health sector and a modified societal perspective are presented as reference case analyses as recommended by the Second Panel on Cost-effectiveness in Health and Medicine [30].

## Methods

### Trial design, setting and sample

The study was a single-blinded parallel group randomised controlled trial undertaken with childcare services within New South Wales (NSW), Australia. Details of the trial protocol have been published elsewhere [31]. In summary, the sample of 54 services was drawn from a pool of 252 long day care services in NSW that both provided food to children in care and were current clients of a single specific childcare management software (CCMS) provider. The intervention involved access to a web-based menu planning and decision-support tool.

Eligible services had to meet the following requirements: (i) be open for ≥ 8 h each weekday; (ii) prepare and provide at least one main meal and two snacks to children onsite each weekday; (iii) have autonomy to make menu planning decisions within the service and; (iv) have a staff member responsible for menu planning (menu planners) with sufficient English to engage with the intervention. Excluded services were those that outsourced menu planning, did not cater for children aged 3–6 years, catered exclusively for special needs children, or were run by the NSW Department of Education and Communities. Twenty-seven services were randomised to intervention and 27 services to the control group.

The trial was prospectively registered with the Australian New Zealand Clinical Trials Registry (ACTRN12616000974404) and approved by the Hunter New England (approval no: 16/02/17/4.05) and the University of Newcastle (approval H-2016-0111) Human Research Ethics Committees.

### Economic study

A prospective, trial-based economic evaluation of the intervention versus usual practice was conducted from both health care sector and modified societal perspectives over a 1-year time horizon, consistent with the length of the trial (12 months). All costs were calculated and reported in $AUD, 2017/18. The modified societal perspective was constrained to those who would be impacted financially by the intervention, health care providers and childcare services.

### Usual practice (control)

Relevant international, national and state guidelines recommend that childcare services implement evidence-based practices to improve the provision of healthy food [32–34]. Childcare services in Australia that provide food to children are required by national accreditation standards to serve foods consistent with the Australian Dietary Guidelines (ADG) [35]. In NSW, the Caring for Children [34] resource outlines best practice dietary guidelines for the childcare sector, which are consistent with the ADG. Assessment and compliance officers in NSW regulate service accreditation and use the Caring for Children guidelines to determine if services meet accreditation standards in relation to dietary guidelines for the sector. Services in NSW are also required by law to list all food served to children whilst in care on menus and to make these menus publicly available. In this context, usual practice for childcare services across NSW comprises support from a health promotion officer employed by the local health district to implement the NSW state-wide obesity prevention programme for early childhood, Munch and Move [36]. Support is typically provided upon request from the childcare service, via telephone or face-to-face contact, with referral to supporting resources. For menu planning, support may also be in the form of a menu review and provision of feedback on compliance with dietary guidelines. Childcare services that were randomly allocated to the control group did not receive access to the directed implementation support strategies described below.

### Menu planning support (intervention)

The 12-month intervention targeted menu planners and nominated supervisors within each childcare service. The intervention comprised access to a web-based menu planning tool, titled *feedAustralia*, online resources, online reminders and feedback, as well as training and support for menu planners and nominated supervisors to use the programme. The intervention was informed by previous research where more traditional modes of implementation support, such as printed resources, were trialled with limited success and uncertain cost-effectiveness [15]. In this trial, greater effect, sustainability and cost-effectiveness were anticipated by using a technology platform and online resources. Details of the components in this intervention are detailed in the original effectiveness study [31]. In brief, the intervention comprised of the following: a web-based menu planning programme with decision support including automated menu planning, audit and feedback; online resources and reminders; communication strategies and managerial support; training and support to use the programme and a portable computer tablet.

### Identification, measurement and valuation of trial outcomes

The aim of the implementation intervention was to increase menu compliance with recommended dietary guidelines for childcare services in the state (Caring for Children resource [34]). These guidelines require services to provide daily serves for each of the following food groups: (1) vegetables and legumes/beans (two serves); (2) fruit (one serve); (3) whole grain cereals, foods and breads (two serves); (4) lean meat and poultry, fish, eggs, tofu, seeds and legumes (3/4 serve); (5) milk, yoghurt, cheese and alternatives (one serve); and (6) no ‘discretionary’ foods that are high in energy and low in nutrients (zero serves). Compliance was defined as the provision of the recommended number of serves for that food group per child per day over a 1-week period.

The primary trial outcome was defined as the mean number of food groups compliant with dietary guidelines: Compliance of the menu with nutrition guidelines was assessed using best practice assessment methods, based on calculation of the serves of each food group provided per child, per day. The assessment was conducted by a blinded dietitian who randomly selected 1 week of each services’ current menu cycle for detailed menu review. At baseline, 1 week for the current menu cycle was randomly selected during the recruitment phone call for those services that consented. For follow-up, 1 week for the current menu cycle was again randomly selected (approximately 12 months later). The dietitian obtained all recipes, quantities of food served and number of children attending each day to enable detailed calculation of serves of food groups. The mean number of compliant food groups per service (a score out of six) was compared between intervention and control groups at 12 months follow-up.

Secondary outcomes were:

(i) Compliance with guidelines for all food groups: The proportion of services compliant for all of the six food groups was compared between the intervention and control group as assessed via 1-week menu review at baseline and 12 months follow-up.
(ii) Individual food group compliance with dietary guidelines: The proportion of services compliant with dietary guidelines for each of the six individual food groups was compared between the intervention and control group as assessed via 1-week menu review at baseline and 12 months follow-up.
(iii) Mean servings of individual food groups: The mean number of serves for each of the six food groups provided on the menu was compared between the intervention and control groups as assessed via 1-week menu review at baseline and 12 months follow-up.

### Identification, measurement and valuation of resource use

Micro-costing was used to calculate the incremental costs of the intervention compared to usual practice. Specific cost components, assumptions and sources of unit costs are provided in Table 1. Resource use associated with the execution of the intervention was prospectively collected using a customised cost data capture template, designed by health economists from the Hunter Medical Research Institute and compiled by the health promotion officers delivering the intervention. Categories of cost included labour, materials, joint costs where costs are shared across multiple programmes and miscellaneous. Resource use was tagged according to stakeholder expense (public health, childcare service, other).

**Table 1:** Micro costing assumptions and sources of unit costs

Cost component	Trial setting	
	Details and assumptions	Source of unit costs
**Local health district costs**		
Labour		
Face to face training	Health service-funded dietitians and project officers employed to support childcare centres to be compliant with dietary guidelines	Wage rates HSM level D midpoint of $42.50 per hour including 30% on-costs
Support phone calls, newsletter and emails		
Optional online training offered to cooks		
Materials		
Tablets to enable portable access to web-resources	Tablets: Samsung Galaxy tablets provided to intervention centres	Tablets: Exact cost from project records $308.44 average 2016/2017 cost
Stationery—printed resources	Printing: 2 colour pages	Printing: Exact cost from project records $0.10/0.65 per page Officeworks
B&W/colour	2 colour pages	
Blank service action plan (colour)	17 colour pages	
Certificate of participation in training	4 colour pages	
Nutrition training manual	2 B&W pages	
Support Officer Action Plan examples	21 colour pages	
Training evaluation questionnaire		
FeedAustralia User Guide (colour)		
Phone calls	Follow-up phone calls (3 per service) Online training phone calls (additional to follow-up phone calls)	Exact cost from project records $0.30 Telstra local rate
Travel and expenses	Meals, travel and accommodation costs	Exact cost from project records Travel by car valued using the NSW Treasury ‘TC17-10 Meal, Travelling and Related Allowances’ monetary rates per kilometre travelled [37]
**Childcare centre costs**		
Labour	Self-reported time in hours collected via baseline and follow-up using telephone surveys	Fair Work Australia award wage rates per hour [31] (lower, midpoint, upper) adjusted to include 30% overheads Cook ($26.13, $28.65, $32.25) Educator ($30.56,$33.75, $34.26) Supervisor ($35.28, $35.80, $36.44) Director L1 ≤ 39 children ($40.69, $43.26, $44.71) Director L2 40-59 children ($41.20, $43.65, $45.21) Director L3 ≥ 60 children ($41.70, $44.19, $45.72)
Cook		
Supervisor		
Educator		
Director		

Resource use data associated with the labour time spent undertaking menu planning and reviewing was collected for both intervention and usual practice services at baseline and follow-up. These data were collected using survey instruments employed in the trial and completed by nominated supervisors and menu planners within each of the childcare services via telephone. Baseline and follow-up surveys included questions on how much money the services usually spent on buying food and drinks for children per month.

Labour costs, including opportunity costs incurred during the intervention uptake by childcare service staff (cooks, educators, supervisors and directors), were captured in the form of staff time (hours) and valued using the midpoint from relevant Australian wage rate ranges [38]. All other resource use categories were valued using market rates. All costs were reported in 2017/18 Australian dollars ($AUD).

### Economic analysis

The costing analysis was undertaken from both health sector and modified societal perspectives. All analyses were carried out using the Microsoft Excel software Office 365. The direct health sector cost to support uptake of the web-based menu planning intervention was calculated. Costs to the childcare services in each study arm to undertake menu planning and reviewing were also calculated. The incremental cost of the intervention was calculated as the net difference in the cost to undertake menu planning and review (essential elements to planning healthier menus that meet guidelines) plus the direct cost of the intervention.

Owing to the complex set of outcomes included in the effectiveness study, the evaluation included both CCA and CEA. The cost-consequence analysis reports the incremental cost of executing the intervention alongside the primary and secondary outcomes by way of a score-card. Cost-effectiveness analysis was also conducted to assess the productive efficiency of the intervention compared to usual practice. However, the absence of an explicit willingness to pay threshold for trial-specific cost-effectiveness results can make interpretation of the incremental cost-effectiveness ratio (ICER) difficult. A recent method of generating a threshold to aid decision making was published by Hyewon and Levine [39]. In this method, the cost and outcomes associated with usual practice, combined in an average cost-effectiveness ratio (ACER), are assumed to represent an implicit willingness-to-pay threshold, having already been implemented by the health care system or society. A relative value index (RVI) is calculated by dividing the usual practice ACER by the ICER calculated for the new intervention. The decision rule follows that if the RVI is greater than 1 the intervention is offering additional outcomes at an ‘acceptable’ cost and should be implemented. That is, the incremental cost per unit increase in compliance score with the web-based intervention is lower than the cost per level of compliance attainable with usual practice.

Cost-utility analysis, an alternate method of economic evaluation where intervention effects are measured in terms of impact on length of life and impact on quality of life (utility) summarised as quality-adjusted life years (QALYs), was not selected in this study for the reason that the underlying trial was an implementation trial. This trial was appropriately focussed on the measurement of compliance as the implementation outcome, as opposed to final health outcomes.

### Handling of bias and missing data

To avoid bias in the economic analysis [40], any baseline differences in cost between the groups were adjusted by calculating trimmed or truncated means. Similarly, it is important not to ignore missing data. Inappropriate handling of missing data can lead to misleading inferences in economic evaluations [41]. While cost-effectiveness analyses conducted alongside trials are an important source of information for decision makers, trials rarely succeed in collecting all the required information [42]. Guidance for handling missing data in trial-based cost-effectiveness analyses and for treating missing cost data specifically recommends multiple imputation [43–45]. In this evaluation, the treatment of missing data was handled using a combination of methods: multiple imputation using linear regression models and quantile modelling.

### Uncertainty and sensitivity analyses

To account for uncertainty due to sampling variation, nonparametric bootstrapping analysis with 2000 iterations was used. The bootstrapped ICERs were graphically mapped on a cost-effectiveness plane and used to derive a cost-effectiveness acceptability curve (CEAC) indicating the probability of the intervention being cost-effective at various levels of society’s willingness to pay per unit change in outcome [26]. The willingness to pay threshold was informed by the ACER calculated for usual practice [46].

Sensitivity analysis is used to illustrate and assess the level of confidence that may be associated with the conclusion of an economic evaluation [47]. In this study, we undertook several one-way sensitivity analyses. First, on the basis that labour time in menu planning and review was the largest driver of cost, we examined the robustness of results to changes in the value of the wage rates for childcare service staff involved in menu planning and review. In the base case analysis, the midpoint of rate ranges were used. In the sensitivity analysis, the upper and lower rates were used [38]. In the second sensitivity test, we conducted complete case analysis, including only those variables with no missing data for both intervention and usual practice services. Third, we adjusted for missing data making the less conservative assumption that the proportional change in childcare service cost for the known observations would apply to the missing data for both intervention and usual practice services.

## Results

The impact of the intervention on the specified trial outcomes are presented in detail elsewhere [37]. Compared to usual practice services, intervention services achieved a non-significant increase in the primary outcome; the mean number of food groups on the menu that were compliant with dietary guidelines (relative effect size 0.26; 95% CI − 0.61 to 1.14; *p* = 0.55).

Secondary outcomes were as follows: No service in either the intervention or usual practice arms were compliant with all six of the food groups at baseline, and none of the services in either group achieved compliance at 12 months follow-up. There were no significant differences in compliance between groups for any of the six individual food groups at 12 months: vegetables (OR 0.37; 95% CI 0.01–10.82; *p* = 0.56), fruit (OR 2.46; 95% CI 0.41–14.58; *p* = 0.32), cereals (OR 1.21; 95% CI 0.2–7.51; *p* = 0.83), meat (OR 1.7; 95% CI 0.14–20.56; *p* = 0.68), dairy (OR 0.97; 95% CI 0.18–5.18; *p* = 0.97) and discretionary foods (OR 0.99; 95% CI 0.06–17.29; *p* = 0.99). Significant increases were achieved in the mean servings of fruit (ES 0.21; 95% CI 0.02–0.4; *p* = 0.03) and a significant reduction in the mean number of times per week that discretionary foods were provided (ES − 0.33; 95% CI − 0.54 to − 0.11; *p* = 0.003).

The mean direct cost of implementing the intervention was calculated to be $1013 (95% UI $978, $1051) per service including labour time and materials (from the local health service perspective).

At baseline, intervention services were calculated to spend a mean cost of $7094 per year on activity related to menu planning and review, compared to an adjusted trimmed mean of $8606 for usual practice services.

The proportion of missing 12-month follow-up data pertaining to the self-reported costs of menu planning and reviewing was 37%. These data were missing due to differences between the baseline and follow-up surveys. There was also a poor response rate (40%) by services providing data on food and drink expenditure. These data were requested to be able to assess if the intervention had any direct financial consequences. The data that was provided showed variation in the baseline amount spent on food and drink per child (mean $2.56, range $0.8–$5.00). From the follow-up data provided, it was not possible to determine if the intervention had resulted in changes to food and drink expenditure.

Compared to usual practice services, intervention services spent less labour time on menu planning and reviewing both prior to the commencement of the study and at 12 months follow-up (Table 2).

**Table 2:** Resource utilisation and cost by study group

	Usual practice mean, 95% UI	Intervention mean, 95% UI
Intervention delivery cost	$0	$1013 ($927, $1096)
Childcare centre labour cost of menu planning and reviewing (Baseline)	$8606^a^	$7094
Childcare centre labour cost of menu planning and reviewing (Follow-up)	$7640	$4634
Incremental difference (95% UI)	− $482 (− $1008, − $57)	

^a^Trimmed mean

The linear regression modelling included a variety of alternate models reflecting (a) no adjustments, (b) adjustments using trimmed annual costs for baseline differences and (c) imputation of missing values with percentage difference from non-missing outcomes (but no trimming). We also used quantile regression to examine follow-up yearly costs allowing for a conditional distribution. Quantile regression is a regression model in which a specified conditional quantile of the outcome (follow-up yearly costs) is expressed as a linear or nonlinear function of the covariates in the model. The model was estimated at the 0.10, 0.25, 0.50, 0.75 and 0.90 quantiles. Wald tests were implemented to test results of heteroskedasticity for each covariate across each quantile.

At 12 months follow-up, these costs were calculated to be $4634 for intervention services and $7640 for usual practice services. The mean difference in total cost (intervention plus menu planning and review investment) was − $482 (95% UI − 859, − $56). That is, the average cost per intervention service would be $482 less per year than for usual practice services.

### Cost-consequence analysis (CCA)

The results of the cost-consequence analysis are displayed in Table 3. Costs associated with the intervention and usual practice are presented as mean costs with 75% uncertainty intervals derived from the bootstrapped samples. As outlined above, costs were calculated using a bottom-up approach. Consequences were included that represent the primary and secondary study outcomes: mean compliance score, individual food group compliance and mean servings of individual food groups. Mean outcomes are presented alongside the bootstrapped generated uncertainty intervals.

**Table 3:** Summary of costs and consequences

	Usual practice mean, 95% UI	Intervention mean, 95% UI	Difference mean, 95% UI
**Costs**			
Intervention cost	$0 ($0, $0)	$1013 ($927, $1096)	$1013 ($927, $1096)
Childcare centre cost of menu planning and review	− $966 (− $1339, − $564)	− $2460 (− $3320, − $1653)	− $1494 (− $2755, − $313)
Total cost			− $482 (− $1008, − $57)
**Consequences**			
Primary outcome result: non-significant increase in the mean number of food groups that were compliant with guidelines			
Compliance scores	0.33 (-0.22, 0.93)	0.52 (-0.074, 1.22)	0.19 (0.15, 0.30)
Secondary outcome results:			
(1) Non-significant increases in compliance for food groups: fruit, dairy and alternatives and discretionary			
Individual food group compliance scores			
Fruit	0 (− 0.26, 0.26)	0.15 (0, 0.32)	0.15 (0.04, 0.22)
Vegetables	0.15 (0, 0.30)	0.04 (− 0.07, 0.19)	− 0.11 (− 0.15, − 0.07)
Cereals and breads	− 0.07 (− 0.26, 0.15)	− 0.07 (− 0.30, 0.15)	0 (− 0.04, 0.04)
Meat and alternatives	0.15 (− 0.11, 0.41)	0.15 (− 0.07, 0.41)	0 (− 0.04, 0.04)
Dairy and alternatives	0.04 (− 0.07, 0.19)	0.11 (− 0.04, 0.26)	0.07 (0.04, 0.11)
Discretionary	0.07 (− 0.07, 0.22)	0.15 (0, 0.33)	0.07 (0.04, 0.11)
(2) Significant improvements in mean servings for fruit and discretionary food groups and non-significant changes in mean servings for vegetables, cereals and breads and meat and alternatives			
Individual food group mean number of servings:			
Fruit	− 0.01 (− 0.37, 0.35)	0.28 (− 0.06, 0.62)	0.29 (0.27,0.31)
Vegetables	− 0.08 (− 0.73, 0.54)	0.7 (0.19, 1.24)	0.77 (0.67, 0.92)
Cereals and breads	− 0.20 (− 0.91, 0.53)	0.14 (− 0.37, 0.66)	0.34, (0.09, 0.54)
Meat and alternatives	− 0.17 (− 0.44, 0.07)	0.21 (0.06, 0.39)	0.38 (0.32, 0.49)
Dairy and alternatives	− 0.21 (− 0.53, 0.07)	− 0.30 (− 0.68, 0.02)	− 0.09 (− 0.15, − 0.05)
Discretionary	0 (− 0.48, 0.48)	− 0.48 (− 0.78, − 0.22)	− 0.48 (− 0.70, − 0.30)

### Cost-effectiveness analysis

While the intervention achieved improvements in service menus, the primary outcome of a statistically significant increase in overall compliance of menus with implementation of dietary guidelines was not met. For this reason, the cost-effectiveness analysis included calculation of incremental cost-effectiveness ratios for both the primary outcome and for those secondary outcomes showing significant improvements in the mean number of servings of individual food groups (fruit and discretionary food).

The calculation of a cost saving associated with the intervention coupled with positive effect sizes for the outcomes listed above resulted in dominant ICERs. That is, the intervention is both less costly and more effective than usual practice.

Using the sample data from the usual practice group, the average cost-effectiveness ratio (ACER) for usual practice was calculated to be − $2897. As described above, this ratio constitutes an implicit benchmark threshold and reflects the opportunity cost to implement the intervention [46]. In this analysis, the RVI was calculated to be 1.11 and should be interpreted to mean that the intervention offers greater outcomes at an ‘acceptable’ cost and should be implemented.

### Uncertainty and sensitivity analysis

**Fig. 1:**
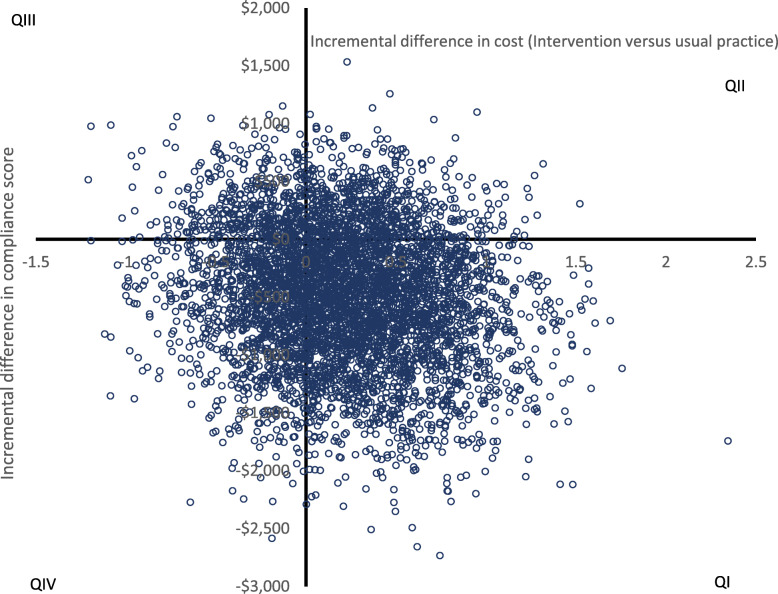
Bootstrapped results on the cost-effectiveness plane for compliance score outcome. Q1: Intervention dominates—more effective and less costly; QII: ICER—more effective with higher costs; QIII: Intervention is dominated—less effective and more costly; QIV: ICER—less effective with less cost

The joint distribution of the incremental costs and compliance effects for the primary outcome measure is shown in Fig. 1.

Fifty-four percent of the bootstrap replicates were located in quadrant I (intervention dominant with less cost and improved compliance), and 78% of the replicates were located across quadrants I and IV (less costly). The result uncertainty characterised by the distribution of the bootstrap replicates reflects variability both in outcome and cost. Such variability is unsurprising given the study was conducted in a diverse population of community-based childcare services.

**Fig. 2:**
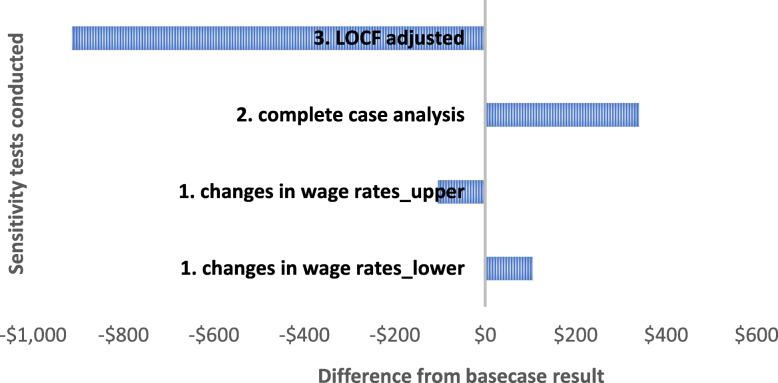
Sensitivity analysis results—difference from base case in mean incremental cost per childcare centre

Three specific one-way sensitivity analyses were conducted. The results are shown in Fig. 2. First, we examined the robustness of results to using alternative (higher and lower) wage rates for childcare service staff involved in menu planning and review. Using the published wage ranges for these roles resulted in a 7% decrease/increase in the childcare service costs at baseline and follow-up. Second, we examined the effect of performing a complete case analysis where all observations with missing data were removed. This resulted in a positive net difference in childcare service cost between intervention and usual practice services. Third, we adopted a less conservative approach and accommodated missing data by adjusting the last observation carried forward cost variables by the proportional change observed in the observable follow-up cost variables. This resulted in the largest cost saving estimate between intervention and usual practice services of $913.

## Discussion

There is dearth of economic evaluations of approaches to improve and sustain the implementation of public health interventions across a range of settings [24]. Specifically, there are no comparable economic evaluations of implementation-interventions in this setting focussed on childhood obesity. More broadly, there are published economic evaluations of early childhood obesity prevention interventions conducted in other countries [48–50], and these studies are typically inconclusive in their assessment of cost-effectiveness given uncertainty in measures of effect.

This study is one of few economic evaluations in the field of implementation science applied to preventive health. For policy makers and practitioners interested in implementation strategies to improve and sustain nutrition in childhood and reduce the risk of obesity amongst children in early childhood, this study addresses an evidence gap and provides information relevant to decision making. While there is uncertainty in the effectiveness of the intervention in increasing the overall compliance of menus with implementation of dietary guidelines in childcare services, significant improvements in the mean servings of individual healthy food groups were found. Effect size uncertainty notwithstanding, economic evaluations should still be conducted because one intervention may be comparatively cost-reducing, as appears to be the case in this study.

Further, the costing analysis conducted within the economic evaluation is vital to inform the future costs to sustain and scale the intervention. Funding stability, establishing a consistent financial programme base, has been reported to be a critical factor in ensuring sustainability [51]. In this study, the approach to identifying, measuring and valuing resource use across the stakeholders allows funding stability to be assessed. A clear strength of the analysis is the prospective nature of the economic evaluation conducted within the RCT study design. This allowed for the resource use data to be more accurately captured from each of the invested stakeholders at both the baseline and follow-up points in the study. Appropriately for trials of implementation-interventions, the analysis was restricted to intermediate outcomes relevant to improving guideline compliance [52]. Overall, the results of this study suggest that it is possible for implementation strategies to deliver economic value even when the intervention outcomes are modest. These results advance economic evaluation of obesity prevention through increasing the efficiency of existing evidence-based policy. However, a number of limitations should also be recognised. First, while the application of CCA to implementation strategies may be appropriate given the range of outcomes in the trial, CCA does not provide a clear decision metric, and without an explicit acknowledgement by decision makers regarding their willingness to pay for increased compliance, or value propositions based on cost-consequence score cards, interpretation of the calculated incremental cost-effectiveness ratio (ICER) is hampered. In this analysis, additional evidence to inform and aid decision making was provided by calculation of the relative value index presented in conjunction with the cost-consequence analysis. Second, the data collection regarding childcare service labour time spent conducting menu planning and review relied on self-reports from the services. Despite randomisation, there was a difference between study arms in the childcare service costs at baseline. The difference was attributed to one outlier in the usual practice group who reported costs that were four standard deviations from the mean and two outliers who reported costs that were two standard deviations from the mean. Since the data were self-reported, it is unclear if these were true differences in time spent conducting menu planning and reviews or if there were errors in reporting. In the analysis, the skewness was accounted for using a trimmed mean to mitigate the effects of the outliers.

Second, as described in the ‘Methods’ section, measurement of the primary and secondary outcomes was constrained to a single week at baseline and a single week at follow-up, selected at random. While compliance was assessed using best practice assessment methods, it is an assumption that the observations made during the single, short time points are representative.

Finally, the economic evaluation was not able to adequately measure any financial consequences such as changes in the acquisition cost of food and drink. Both baseline and follow-up questions were asked of childcare services regarding food budgets, but the data returned was of insufficient quality to be included in the analysis.

Given the lack of economic evidence and uncertainty in the little evidence that does exist, there is an argument for the application of value of information analysis and value of implementation analysis in addition to applied economic evaluation. Value of information analysis provides a monetary value for the potential benefits of research that can be compared with the costs to determine if further research is worthwhile [53, 54]. Value of implementation analysis is a means of identifying the potential value of investing in implementation policies [55]. Taking the results from this economic analysis, the combination of approaches would inform decision making in two ways. First, it would support the decision to invest in implementation or not and second could be used to determine if further implementation trials are warranted [53].

## Conclusion

Robust economic evaluation evidence should form basis of decision making in health. In this study, the trial-based economic evaluation showed that, compared to usual practice, web-based programmes may offer an efficient and sustainable alternative for childcare services to improve the provision of healthy foods to children in their care.

## Data Availability

The research team acknowledges the importance of making research data publicly available. Access to the data from this study may be made available to external collaborators following the development of data transfer agreements.

## Abbreviations

ACER: Average cost-effectiveness ratio
AUD: Australian dollars
CCA: Cost-consequence analysis
CCMS: Childcare management software
CEA: Cost-effectiveness analysis
CEAC: Cost-effectiveness acceptability curves
CI: Confidence interval
ICER: Incremental cost-effectiveness ratio
LOCF: Last observation carried forward
NSW: New South Wales
RCT: Randomised controlled trial
RVI: Relative value index
UI: Uncertainty interval
